# Stereotactic radiosurgery planning of vestibular schwannomas: Is MRI at 3 Tesla geometrically accurate?

**DOI:** 10.1002/mp.12068

**Published:** 2017-02-16

**Authors:** M. A. Schmidt, E. J. Wells, K. Davison, A. M. Riddell, L. Welsh, F. Saran

**Affiliations:** ^1^ The Institute of Cancer Research CR‐UK & EPSRC Cancer Imaging Centre The Royal Marsden NHS Foundation Trust Sutton UK; ^2^ Medical Physics Royal Marsden NHS Foundation Trust London UK; ^3^ Radiology Department Royal Marsden NHS Foundation Trust London UK; ^4^ Neuro‐Oncology Unit Royal Marsden NHS Foundation Trust London UK

**Keywords:** distortion, MRI, Stereotactic Radiosurgery

## Abstract

**Purpose:**

MRI is a mandatory requirement to accurately plan Stereotactic Radiosurgery (SRS) for Vestibular Schwannomas. However, MRI may be distorted due not only to inhomogeneity of the static magnetic field and gradients but also due to susceptibility‐induced effects, which are more prominent at higher magnetic fields. We assess geometrical distortions around air spaces and consider MRI protocol requirements for SRS planning at 3 T.

**Methods:**

Hardware‐related distortion and the effect of incorrect shimming were investigated with structured test objects. The magnetic field was mapped over the head on five volunteers to assess susceptibility‐related distortion in the naso‐oro‐pharyngeal cavities (NOPC) and around the internal ear canal (IAC).

**Results:**

Hardware‐related geometric displacements were found to be less than 0.45 mm within the head volume, after distortion correction. Shimming errors can lead to displacements of up to 4 mm, but errors of this magnitude are unlikely to arise in practice. Susceptibility‐related field inhomogeneity was under 3.4 ppm, 2.8 ppm, and 2.7 ppm for the head, NOPC region and IAC region, respectively. For the SRS planning protocol (890 Hz/pixel, approximately 1 mm^3^ isotropic), susceptibility‐related displacements were less than 0.5 mm (head), and 0.4 mm (IAC and NOPC). Large displacements are possible in MRI examinations undertaken with lower receiver bandwidth values, commonly used in clinical MRI. Higher receiver bandwidth makes the protocol less vulnerable to sub‐optimal shimming. The shimming volume and the CT‐MR co‐registration must be considered jointly.

**Conclusion:**

Geometric displacements can be kept under 1 mm in the vicinity of air spaces within the head at 3 T with appropriate setting of the receiver bandwidth, correct shimming and employing distortion correction.

## 1 Introduction

Planning stereotactic radiosurgery (SRS) frequently requires the co‐registration of magnetic resonance imaging (MRI) and computed tomography (CT) examinations; the excellent soft‐tissue contrast of MRI permits optimal delineation of the gross tumor volume (GTV) and organs at risk (OAR), while CT contributes the electron density data and bone anatomy contrast.^1^ CT examinations can be safely assumed to be geometrically accurate, but for MRI the geometrical accuracy can be compromised by imperfections in the main magnetic field and in the gradient fields, and by the distribution of magnetic susceptibility values within biological tissues.^2^, ^3^ These geometrical distortions are particularly relevant to SRS planning, as treatment is often delivered in either a single or a small number of fractions (typically 3 to 5) and dose gradients can be very high.^4^ MRI is a mandatory requirement to accurately plan SRS for Vestibular Schwannomas (VS),^5^ and steep dose gradients are often prescribed to preserve hearing and to prevent facial nerve injury.^6^, ^7^, ^8^, ^9^

Many of the intracranial tumors treated with SRS abut air spaces, such as the mastoid sinus cavity and the internal auditory canal (IAC), in regions affected by susceptibility‐related magnetic field inhomogeneity.^10^ MRI at 3 T is well established in clinical neuroscience, and provides the advantage of a higher signal‐to‐noise ratio (SNR).^11^, ^12^ However, susceptibility‐related field inhomogeneity is also known to increase at higher field strengths, as biological material becomes increasingly magnetized.^13^ As a result, it is currently unclear whether the geometrical accuracy of MRI examinations performed at 3 T is sufficient for SRS treatment planning in the vicinity of air spaces. Here, we investigate the geometrical distortions within anatomical MRI examinations of the head at 3 T on a standard clinical MRI system, and consider scanning protocol requirements for SRS treatment planning by focusing on Vestibular Schwannomas and other lesions adjacent to air spaces within the head. Our aim was to restrict the geometrical displacements to no more than 1 mm over the volume of interest.

## 2 Methods

### 2.A. Data acquisition

This work was approved by the Research Ethics Committee; subjects were scanned at 3 T (MAGNETOM Skyra, Siemens Medical Systems, Erlangen, Germany), after written consent. Three separate potential sources of geometric distortion were investigated: hardware imperfections (non‐uniform static magnetic field and gradient fields), shimming errors, and field inhomogeneity associated with the subjects’ own distribution of magnetic susceptibility values.

Firstly, the hardware‐related geometric distortions were investigated with a previously described 3D structured test object, a three‐dimensional array of tubes (400 × 400 × 250 mm^3^)^2^. High‐resolution 3D T1‐weighted MRI was performed (TE/TR = 1.3/3.5 ms, 485 Hz/pixel, 0.89 × 0.89 × 1 mm^3^ resolution). The manufacturer's own automated shimming routines and post‐processing for 3D distortion correction were employed. Displacements from the true position were estimated over a volume that approximates the volume of the human head (220 × 220 × 220 mm^3^). MR and CT images of this test object were registered (rigid registration) in a treatment planning system for this purpose (Pinnacle³ 9.8, Philips).

To investigate the effect of shimming gradient on distortion, a spherical test object (17 cm diameter) was scanned (TE/TR/TI = 1.58/1070/900 ms, Flip Angle 8°, 890 Hz/pixel, approximately 1 mm^3^ isotropic), both with the automated shimming over the entire object volume, and with the shimming currents manually adjusted to apply the maximum linear gradient field (1750 *μ*T/m), now referred to as “shimming gradient.” The maximum shimming gradient was applied to each axis in turn and the central frequency was re‐adjusted manually. Three separate data sets were acquired and compared to the original images acquired using automated shimming. Displacements were measured at the surface of the sphere.

Next, high‐resolution magnitude and phase images were acquired for field mapping on five volunteers after automated shimming over the entire head volume (TE1/TE2/TR = 2.46/7.38/12 ms, 890 Hz/pixels, approximately 1 mm^3^ isotropic sagittal 3D acquisition, standard head coil). The phase images were subtracted and unwrapped to produce field maps (in‐house software, IDL 8.2, Boulder, CO, USA and Mathworks, Natick, MA, USA).^14^, ^15^, ^16^ The total range of magnetic field variation was assessed over different volumes: (i) the whole head (adjusted for each case), (ii) a volume of 76 mm (S/I) × 76 mm (A/P) × 60 mm (L/R) centered over the ear canal (left and right side measured separately) and (iii) a volume comprising both the nasopharynx and the oropharynx: 48 mm (L/R) × 64 mm (A/P) and adjusted in length (S/I) for each individual (nasal cavity to epiglottis).

In addition, field mapping was also performed on the uniform 17 cm diameter spherical test object with the readout gradient both in the superior/inferior and inferior/superior direction using the same sequence employed in the volunteer studies. The inversion of the readout gradient allows evaluation of the effect of short‐term eddy currents on field map accuracy: in the absence of any eddy‐current effects, both field maps would be identical. Any differences are thus attributed to the effect of short‐term eddy currents on the field mapping sequence.

As a final direct confirmation of the level of geometrical distortion associated with susceptibility‐related magnetic field inhomogeneity, one volunteer was scanned with two different readout bandwidth values (500 and 890 Hz/pixel) for otherwise identical sequences, using the same shimming. These images were compared, and for these circumstances any differences can be attributed to susceptibility‐related field inhomogeneity.

#### 2.A.1. Calculation of susceptibility‐related displacements

For a given position **r**, the static magnetic field can be described as B_0 _+ b(**r**), where the term b(**r**) is the susceptibility‐related field inhomogeneity. The associated susceptibility‐related displacement d is known to be inversely proportional to the receiver bandwidth Δf:^13^

$$d=\Delta {x.f}_{0}.\left({b/B}_{0}\right)/\Delta f$$

where Δx is the pixel size, f_0_ is the Larmor frequency and Δf is the receiver bandwidth per pixel. Therefore, if the range of magnetic field values within a given volume is known, the maximum displacement of any voxel from its true position due to susceptibility‐related field inhomogeneity can be calculated for a given set of pulse sequence parameters. The displacements in images acquired using the SRS planning sequence and the field mapping sequence can be related to each other directly, as both have the same spatial resolution and pixel bandwidth.

## 3 Results

**Figure 1:**
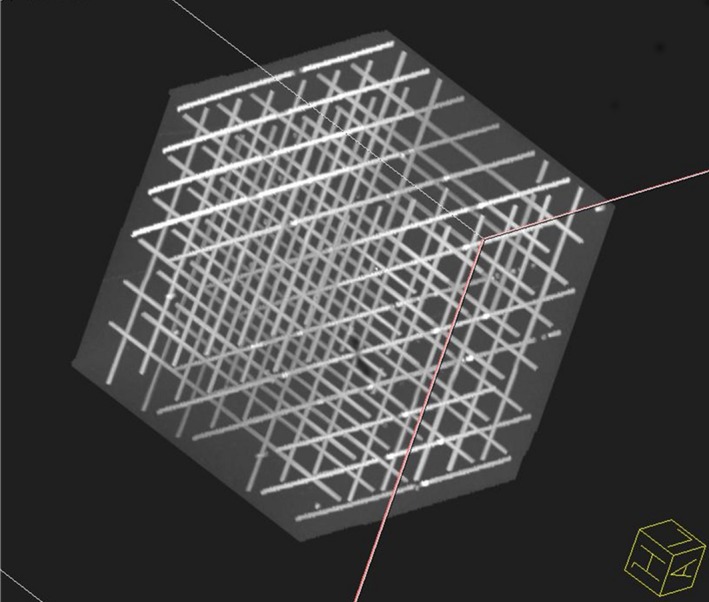
Central portion (220 × 220 × 220 mm^3^) of the structured Linear Test Object, rendered in 3D, after application of the 3D distortion correction software provided by the MRI manufacturer. All lines appear straight. The displacement is estimated not to exceed half of the voxel size (0.45 mm). [Color figure can be viewed at http://wileyonlinelibrary.com]

Figure 1 shows the central portion of the Linear Test Object images; there were no visible displacements in this central volume, all tubes appear straight, and the geometrical distortions are minimal. Considering the limitations of direct measurements on fused MR‐CT data sets, displacements were estimated to be less than half of the voxel size (i.e., less than 0.45 mm), a level consistent with expected imperfections in the test object construction.

**Figure 2:**
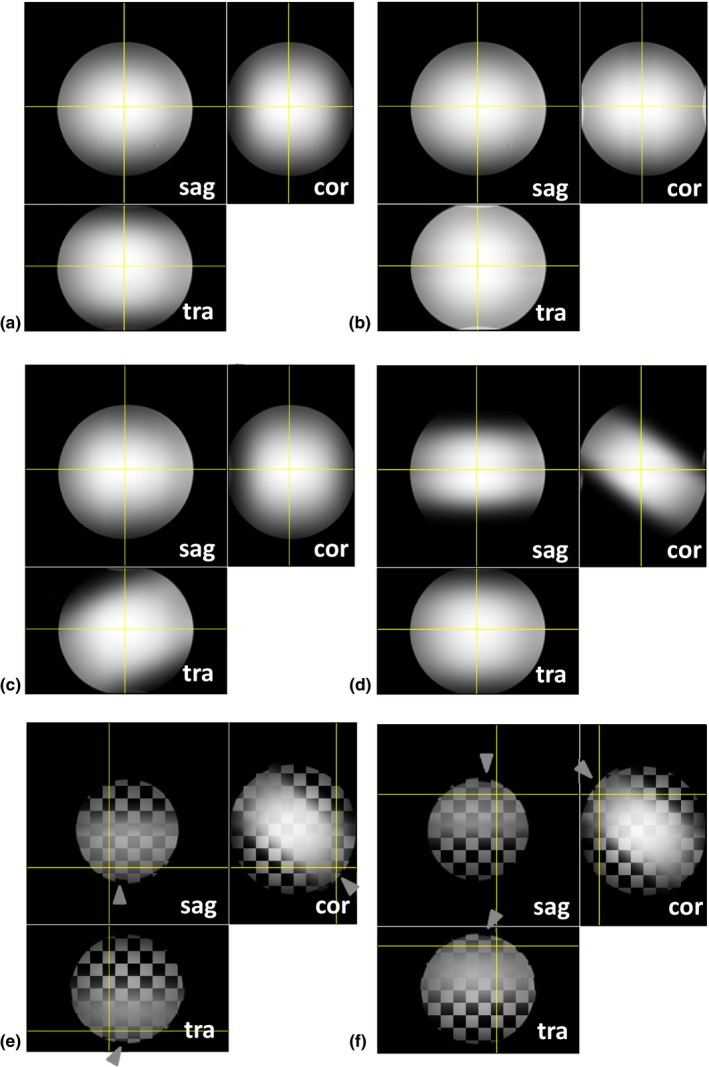
17 cm diameter uniform test object showing the effect of linear gradients associated with shimming errors. (a) Correct shimming. (b) Additional shimming gradient in the slab selection direction changes the thickness of the slab. (c) and (d) Additional shimming gradients along the phase encoding and readout direction cause rotation of the selected slab. Additional gradient parallel to the readout direction causes geometrical distortion. The correct shimming (2a) and the incorrect shimming of 2d are combined in (e) and (f). The overall displacement (arrows) reaches 4 mm along the readout direction. [Color figure can be viewed at http://wileyonlinelibrary.com]

Figure 2 shows images of the spherical test object and the effect of large shimming gradients along the three main axes. These gradients interfered with the slab selection process. The slab thickness changed when the shimming gradient was orientated along the slab selection direction: a slightly thicker slab caused some wrapping (Fig. 2b). The orientation of the slab changed when the shimming gradient was along the phase encoding and readout direction; the slab rotation was 22° and 55° in Figs. 2c and 2d, respectively. The shimming gradient did not distort the phase‐encoding process, but distorted the images when added to the readout gradient. The associated distortion was greatest with the shimming gradient along the readout direction; a change in scale was visible in Figs. 2e and 2f, and the displacement reached 4 pixels (4 mm) at the surface of the sphere.

**Figure 3:**
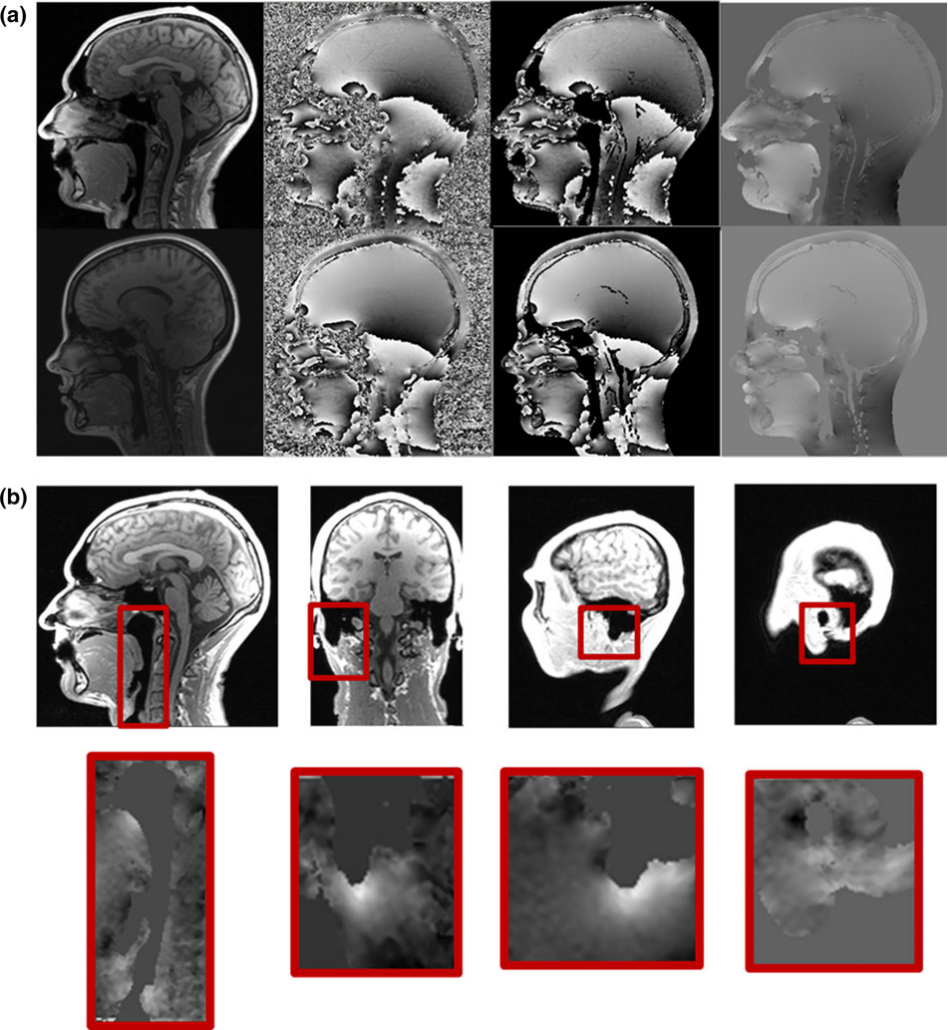
(a) Images of 2 volunteers showing similar patterns of magnetic field inhomogeneity. Gradient over neck is present in all subjects. From the left: magnitude image, field map, thresholded field map, unwrapped field map. (b) Field map details show areas most affected by susceptibility‐related field inhomogeneity surrounding air spaces, showing both higher and lower magnetic field values in close proximity. [Color figure can be viewed at http://wileyonlinelibrary.com]

For the five volunteers, the range of magnetic field values found over the head volume was 3.2 ± 0.2 ppm (mean ± standard deviation, from 3.0 to 3.4 ppm). In all five subjects a macroscopic gradient was observed along the superior/inferior direction on the neck (Fig. 3a). Steep field gradients were detected adjacent to air spaces, as expected (Fig 3b). These gradients occur towards the air spaces and are located within 4–5 mm of the tissue‐air interface. The highest and lowest magnetic field values measured within the IAC regions and within the naso‐oro‐pharyngeal region were located adjacent to air spaces in all cases. The range of measured magnetic field values was 2.3 ± 0.3 ppm and 2.3** **± 0.2 ppm for the internal auditory canal (left and right, from 2.0 to 2.7 ppm and from 1.9 to 2.8 ppm, respectively) and 2.3 ± 0.3 ppm for the naso‐oro‐pharynx (range 1.9 to 2.9 ppm).

Field maps of the same spherical test object obtained with the readout gradient in the superior/inferior and inferior/superior direction were practically identical. After subtraction, the standard deviation of the phase values was 0.18 radians, and the errors in field mapping attributed to eddy currents are thus below 0.05 ppm over the central volume (17 cm diameter).

The largest displacement from the correct position expected with either the field mapping sequence or the SRS treatment planning sequence (890 Hz/pixel, 1 mm pixel dimension) was therefore 0.5 mm for the whole head, 0.4 mm for the IAC and 0.4 mm for the naso‐oro‐pharynx. These displacements are inversely proportional to the receiver bandwidth, and could easily be larger than 1 mm for lower bandwidth values. Receiver bandwidths as low as 250 Hz/pixel are routinely used in clinical MRI, and this would lead to 1.7 mm as the largest displacement over the whole head, and 1.4 mm around the air cavities.

**Figure 4:**
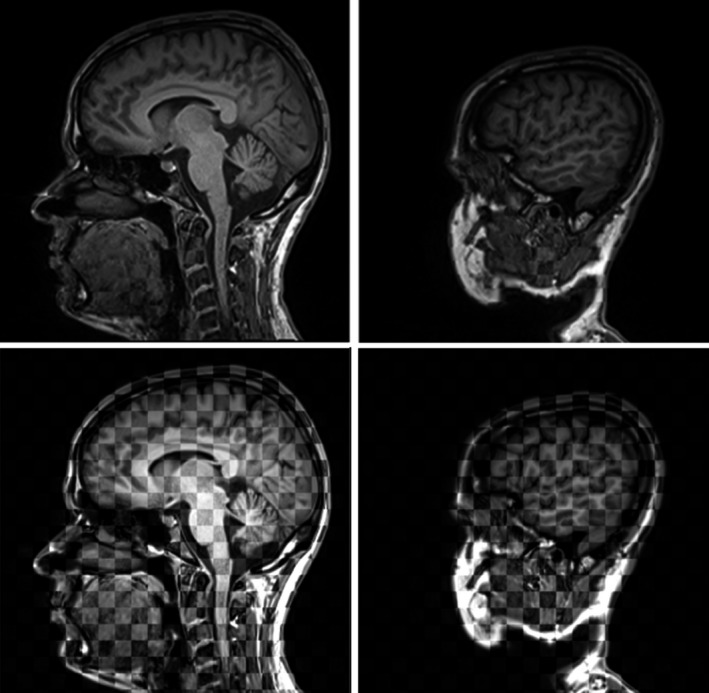
Checker board display of two 3D examinations undertaken within the same conditions but with different bandwidths (500 Hz/pixel and 890 Hz/pixel), after co‐registration. The two examinations are practically identical over the ear canal and the rigid skull. In the top row both data sets use the same gray scale and the checkerboard can only be detected around the oral cavity, where subject motion cannot be excluded. In the bottom row, different gray scales are used for each data set, and the checkerboard is evident.

Figure 4 shows two co‐registered MRI data sets from the same subject acquired with bandwidths of 500 Hz/pixel and 890 Hz/pixel. There are very noticeable differences in the oral cavity, as swallowing motion cannot be prevented over the time taken to acquire the data. However, over the IAC the images appear to be geometrically identical. Geometrical differences within the naso‐oro‐pharynx cannot be attributed to susceptibility effects alone, as the subjects’ tongue and throat cannot be “immobilized.”

## 4 Discussion

In this work the three main sources of geometrical distortion in anatomical MRI scans of the head at high field strength were comprehensively investigated for the naso‐oro‐pharynx and the IAC: hardware characteristics (comprising both static magnetic field and gradient uniformity), susceptibility‐induced field inhomogeneity and shimming. Several studies have considered the use of MRI at high fields (3.0 T and 7.0 T) for SRS planning,^17^, ^18^, ^19^, ^20^, ^21^, ^22^ addressing issues related to the stereotactic frame,^17^, ^18^ which is not used in our MRI examinations. We employ X‐Ray guided Stereotactic Linear Accelerator, which relies on two orthogonal X‐Ray images to locate the anatomy in space. The PTV is outlined using all information available in co‐registered CT‐MR data sets.

Test object measurements demonstrated that the non‐uniformity of the gradient fields is adequately compensated for by the manufacturer's own 3D distortion correction post‐processing software for the central region of the magnet, occupied by the subject's head. The test object design, based on long cylindrical structures,^2^ minimizes susceptibility‐related distortions. Furthermore, the MRI pulse sequence employed for SRS planning makes use of high receiver bandwidth and high readout gradients; under these conditions, the main source of geometrical distortion is expected to be non‐uniform gradients.^23^ The spatial dependence of gradient fields is mainly determined by the design of the gradient coils and is expected to be effectively corrected by the general image post‐processing provided by the MRI manufacturer. Although strictly speaking this issue needs to be revisited for each MRI system, in our experience the main MRI manufacturers use similar technology and the provided correction for gradient non‐uniformity achieves similar results around the isocenter; the literature confirms that system‐related distortions are clinically acceptable at 3.0 T^17^, ^18^, ^20^, ^22^ and at 7.0 T^17^, ^19^, ^21^ in a wide variety of clinical systems. Studies considering longitudinal changes in geometrical distortion in general suggest stability of the distortion pattern.^24^, ^25^, ^26^, ^27^ However, recent technological developments, such as MRI systems suitable for MR‐guided RT, are yet to be evaluated.^28^

This work demonstrates that the shimming coils can produce significant macroscopic gradients which are capable of disturbing the spatial encoding of the signal: this displacement reached 4 mm in test objects, with additional shimming gradients along the readout direction within the 17 cm diameter volume considered. This value is consistent with the maximum linear gradient produced by the shimming coils (1750 *μ*T/m). A shimming error of this magnitude is highly unlikely to occur, this is a “forced error.” If such error occurred, it would be very likely to be noticed by the user, as it would disturb the slab excitation. However, other non‐linear shimming fields can produce distortion patterns which are more difficult to detect by visual inspection. Therefore, our results demonstrate the importance of standardizing the shimming to minimize the probability of shimming errors occurring. From this point of view, shimming over large volumes (the whole head) is safer than attempting to shim over smaller volumes, where magnetic susceptibility‐related field gradients are higher and vary in direction (the IAC only, for example). If the whole skull is going to be used in MR‐CT registration, it would not be advisable to shim over a smaller volume (surrounding the IAC, for example) even if it improves the field inhomogeneity locally and thus reduces distortions over the PTV; such approach would make the MR‐CT registration less accurate by introducing distortion to the MRI when the whole skull is considered. Duchin et al. also considered local CT‐MR co‐registration over the volume of interest, and concluded that such approach is less robust.^19^ Our data suggest that it is also undesirable to include the neck in the shimming volume, as a significant magnetic field gradient over this area was measured in all the tested subjects. Prior knowledge of the co‐registration strategy is required to determine the most appropriate shimming strategy. Shimming and co‐registration should be considered together in MRI examinations undertaken for RT planning.

Considering the skull, there is widespread consensus in the literature that susceptibility‐induced displacements are most relevant in the vicinity of airspaces, and are emphasized at higher field strength.^17^, ^18^, ^19^, ^20^, ^21^ Duchin et al. used transformation matrixes from MR‐CT co‐registration to assess the level of distortion, and only found sub‐millimeter errors.^19^ Wang et al. mapped magnetic field from the top of the head to the bottom of the cerebellum, confirming higher field inhomogeneity surrounding air spaces.^20^ The maximum field inhomogeneity in their data set is 4.46 ppm — a value that is in broad agreement with our worst measurement of 3.4 ppm. Stanescu et al. also studied susceptibility‐related distortions, and employed simulations based on CT to estimate field inhomogeneity; the values reported for field inhomogeneity over the brain (5.68 ppm) were larger than the values we measured for the head, but of the same order of magnitude.^22^ Here we presented measured field inhomogeneity values over the IAC and the naso‐oro‐pharynx for the first time. The parameters that we use for SRS treatment planning scans (890 Hz/pixel, approximately 1 mm^3^ isotropic voxels) are associated with displacements up to 0.5 mm for the field inhomogeneity that we measured. The readout gradient we used is part of a very cautious approach — no differences are seen when the receiver bandwidth is reduced from 890 Hz/pixel to 500 Hz/pixel in Fig. 4, suggesting that any displacements are small compared to the voxel size. We employ robust sequences that are less likely to be affected not only by susceptibility‐induced artifacts but also by sub‐optimal shimming. Increasing the receiver bandwidth (and readout gradient) causes a decrease in SNR, and thus the best compromise must be found for each application.

Our results assume that the field map obtained for the tested subjects is correct, and it is therefore important to scrutinize the field mapping methods chosen. We employed a pulse sequence with bipolar gradient pulses and repeated the field map measurement in a test object after a reversal of the readout gradient. No significant differences were found, and therefore the field map is presumed not to be significantly affected by eddy currents. Wang et al.^20^ repeated the field mapping procedure, and their error assessment thus includes also differences associated with small changes in patient position and orientation in relation to the main magnetic field; they found stable results, suitable for *in vivo* monitoring of field inhomogeneity.^20^ The field maps are also geometrically distorted by the susceptibility‐related field inhomogeneity being mapped, as described by Matakos et al.^29^ In our work, the same bandwidth and spatial resolution were employed in field mapping and in SRS planning scans, and thus displacements reaching 0.5 mm are also expected in the field maps. It is important to highlight that these sub‐millimeter displacements have very little effect on the actual phase values which lead to the magnetic field calculation. The images in Fig. 4 support this conclusion.

We have considered the susceptibility‐related displacements arising from the range of magnetic field values measured within a specific anatomical region. These values can be generalized to any 3 T MRI scanner. This approach does not assume that MRI and CT SRS planning images are correctly co‐registered. The displacements we calculated correspond to the worst case scenario, when either an automated image co‐registration process, or a manual image co‐registration performed by an inexperienced operator, could co‐register correctly the volumes associated with the lowest magnetic fields, thereby maximizing the displacements at the highest magnetic fields (or vice versa). In order to avoid this situation, it is best to prioritize the registration of large structures within the volume of interest against the registration of smaller features, as this would reduce the susceptibility‐related displacements overall. In Fig. 4, both data sets at different bandwidths appear identical over the ear canal, suggesting that at least for this particular case, the susceptibility‐related distortions are very small and hardly perceptible when the images are correctly registered.

Our results demonstrate that by using 3D MRI sequences and the manufacturer's own 3D distortion correction software, it is possible to maintain the geometrical displacements under 1 mm at 3T. However, to ensure optimal imaging for SRS treatment planning the following issues should be carefully considered: (i) the setting of the receiver bandwidth, (ii) the shimming, and (iii) the CT‐MR image co‐registration process. These findings have implications beyond MRI for SRS planning: combined MRI‐LINAC systems are being developed for MR‐guided Radiotherapy^30^, ^31^, ^32^ to be used for the delivery of highly conformal image guided RT radiotherapy techniques (IMRT/VMAT). The demands for high geometrical accuracy in MRI are set to grow, alongside the need for specific quality assurance. Our results will assist in obtaining optimal MRI data for RT planning purposes.

## 5 Conclusion

In conclusion, it is possible to maintain clinically acceptable geometric accuracy in the vicinity of air spaces within the head for MRI at 3 T by using 3D pulse sequences and employing the post‐processing for distortion correction provided by the MRI manufacturer. SRS planning MRI examinations can benefit from the superior image quality achieved at 3 T with appropriate setting of the receiver bandwidth. In order to obtain optimal imaging for SRS planning, there are benefits in considering jointly the shimming and the CT‐MR image co‐registration strategy. These findings have implications for SRS planning and MR‐guided Radiotherapy in general.

## Conflicts of interest

The authors have no relevant conflicts of interest to disclose.
